# Microbial diversity, genomics, and phage–host interactions of cyanobacterial harmful algal blooms

**DOI:** 10.1128/msystems.00709-23

**Published:** 2024-06-10

**Authors:** Lauren E. Krausfeldt, Elizaveta Shmakova, Hyo Won Lee, Viviana Mazzei, Keith A. Loftin, Robert P. Smith, Emily Karwacki, P. Eric Fortman, Barry H. Rosen, Hidetoshi Urakawa, Manoj Dadlani, Rita R. Colwell, Jose V. Lopez

**Affiliations:** 1 Department of Biological Sciences, Guy Harvey Oceanographic Center, Nova Southeastern University, Dania Beach, Florida, USA; 2 U.S. Geological Survey, Caribbean–Florida Water Science Center, Orlando, Florida, USA; 3 U.S. Geological Survey, Kansas Water Science Center, Lawrence, Kansas, USA; 4 Cell Therapy Institute, Kiran Patel College of Allopathic Medicine, Nova Southeastern University, Fort Lauderdale, Florida, USA; 5 Department of Ecology and Environmental Studies, Florida Gulf Coast University, Fort Myers, Florida, USA; 6 CosmosID, Rockville, Maryland, USA; 7 Institute for Advanced Computer Studies, University of Maryland College Park, College Park, Maryland, USA; University of California, Riverside, Riverside, California, USA

**Keywords:** cyanobacteria, blooms, microbial biodiversity, microbial interactions, metagenomics, Lake Okeechobee

## Abstract

The occurrence of cyanobacterial harmful algal blooms (cyanoHABs) is related to their physical and chemical environment. However, less is known about their associated microbial interactions and processes. In this study, cyanoHABs were analyzed as a microbial ecosystem, using 1 year of 16S rRNA sequencing and 70 metagenomes collected during the bloom season from Lake Okeechobee (Florida, USA). Biogeographical patterns observed in microbial community composition and function reflected ecological zones distinct in their physical and chemical parameters that resulted in bloom “hotspots” near major lake inflows. Changes in relative abundances of taxa within multiple phyla followed increasing bloom severity. Functional pathways that correlated with increasing bloom severity encoded organic nitrogen and phosphorus utilization, storage of nutrients, exchange of genetic material, phage defense, and protection against oxidative stress, suggesting that microbial interactions may promote cyanoHAB resilience. Cyanobacterial communities were highly diverse, with picocyanobacteria ubiquitous and oftentimes most abundant, especially in the absence of blooms. The identification of novel bloom-forming cyanobacteria and genomic comparisons indicated a functionally diverse cyanobacterial community with differences in its capability to store nitrogen using cyanophycin and to defend against phage using CRISPR and restriction-modification systems. Considering blooms in the context of a microbial ecosystem and their interactions in nature, physiologies and interactions supporting the proliferation and stability of cyanoHABs are proposed, including a role for phage infection of picocyanobacteria. This study displayed the power of “-omics” to reveal important biological processes that could support the effective management and prediction of cyanoHABs.

**IMPORTANCE:**

Cyanobacterial harmful algal blooms pose a significant threat to aquatic ecosystems and human health. Although physical and chemical conditions in aquatic systems that facilitate bloom development are well studied, there are fundamental gaps in the biological understanding of the microbial ecosystem that makes a cyanobacterial bloom. High-throughput sequencing was used to determine the drivers of cyanobacteria blooms in nature. Multiple functions and interactions important to consider in cyanobacterial bloom ecology were identified. The microbial biodiversity of blooms revealed microbial functions, genomic characteristics, and interactions between cyanobacterial populations that could be involved in bloom stability and more coherently define cyanobacteria blooms. Our results highlight the importance of considering cyanobacterial blooms as a microbial ecosystem to predict, prevent, and mitigate them.

## INTRODUCTION

Cyanobacterial harmful algal blooms (cyanoHABs) are an increasingly recognized problem in freshwater lakes and reservoirs around the world (1, 2). They threaten the health of these ecosystems, causing hypoxia and disrupting food webs with negative impacts on the economy, domestic and wild animals, and public health (1, 3). Cyanotoxins (i.e., microcystins, cylindrospermopsins, saxitoxins, and anatoxins) produced by many species of cyanobacteria that bloom can alter the physiology of the aquatic biota, causing health problems in humans and animals ranging from rashes and gastrointestinal stress to respiratory paralysis and death (4). CyanoHABs occur primarily from nutrient enrichment derived from agricultural, industrial, and urban runoff (5).

Nutrients have been established as a major driver for cyanoHABs and cyanoHAB ecology. However, biological processes and the physical and chemical environment influence nutrient dynamics. The physical (e.g*.,* hydrology, depth, and turbidity) and chemical (e.g*.,* pH, alkalinity, and conductivity) properties of a lake essentially control cyanobacteria access to nutrients, altering nutrient bioavailability and mobilization, as well as exposure to light (6–8). Additionally, blooms are complex microbial ecosystems (9), composed of diverse microorganisms with different metabolic capabilities (10–12). Biological processes, e.g., the microbial nitrogen (N), phosphorus (P), carbon (C), and sulfur (S) cycles, in freshwater and sediment ecosystems are also influential on nutrient bioavailability (9–13). Evaluating these dynamics is important in holistically understanding the effect of nutrients on cyanoHABs.

There is evidence for the role of microbial interactions in cyanoHABs. Shifts in both microbial composition and diversity occur during bloom formation and subsequent succession and decomposition (14–17). Microorganisms form tight associations with the cyanobacteria that bloom (18). They benefit from cyanobacteria-derived organic C (19), and there is evidence of their role in the availability of P, N, S, and growth factors, as well as oxidative stress protection (10, 15, 18, 20–22). Interactions among the cyanobacteria that bloom, as well as between cyanobacteria that do not bloom, also require consideration, both in culture and *in situ*. Temperature, light availability, and nutrient availability are key factors in cyanobacterial succession (5, 23, 24), but cyanobacteria genera or genotypes often co-occur (25–27) indicating coexistence or potential competition. Phages are also important in the dynamics with respect to microbial community structure and biogeochemical cycling (28) and affect bloom decline and microcystin release into the water (29, 30). Warming surface waters and the increased prevalence of extreme weather events are anticipated to affect the physical and chemical properties, as well as biological processes, in freshwater systems (31, 32). Characterizing these interconnected microbial relationships are essential for understanding cyanobacteria blooms and consequential for predicting, preventing, and mitigating future cyanoHABs in freshwater ecosystems.

**Fig 1:**
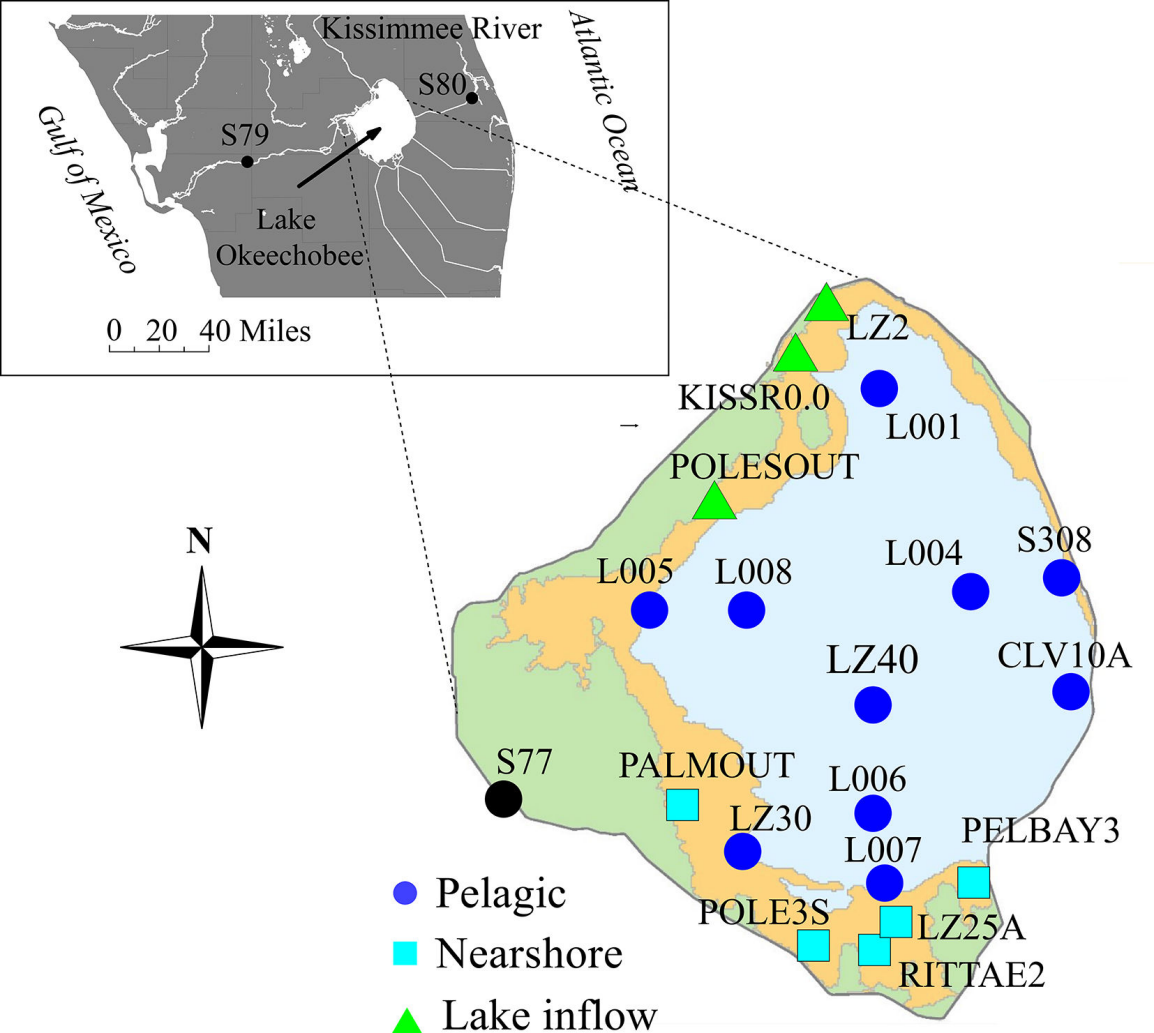
A map of Lake Okeechobee showing ecological zones and the sites sampled in this study. The color of the points corresponds to the ecological zone they represented in this study: dark blue closed circles = pelagic zone, light blue closed squares = nearshore zone, and green closed triangles = lake inflow. The shading on the map represents the boundaries of ecological zones that have been previously described: light blue = pelagic, orange = nearshore, and green = littoral zone. No samples from this study were collected from the littoral zone. The top left shows the relative position of the lake in Florida (USA) and sampling sites S79 and S80. Map of Lake Okeechobee adapted from reference 38 and modified in Adobe Illustrator (39). Inset created using MapMaker by National Geographic (40) and modified in Adobe Illustrator (39).

To address these gaps in knowledge, this study was focused on the microbial ecosystem, notably those community interactions and processes that might be important in cyanobacteria blooms. Lake Okeechobee (Lake O; Fig. 1), the largest lake in the southeastern United States (ca. 14,000 km^2^), was selected as a model ecosystem to study. Lake O is a shallow, heavily managed, eutrophic, and subtropical lake located in South Florida in the United States and is susceptible to the impacts of warming temperatures, high winds, and heavy precipitations associated with tropical storms and hurricanes (33). Recurring and severe cyanoHABs in Lake O have been documented (33) and shown to be dominated by *Microcystis* sp., one of the most common toxic bloom-forming species in lakes around the world (34, 35). *Microcystis* blooms, widespread in Lake O, are linked to the blooms in two waterways receiving most of the outflow from the lake, namely, the Caloosahatchee River Estuary (CRE) and the St. Lucie River Estuary (SLRE), both leading to the Gulf of Mexico and Atlantic Ocean, respectively (33, 35, 36). In 2016 and 2018, the size and intensity of blooms in these rivers were the cause of the State of Florida declaring a state of emergency, based on the severe economic and ecological impacts that expanded to the coastal oceans (35, 37).

## RESULTS

### Microbial community dynamics of Lake O

**Fig 2:**
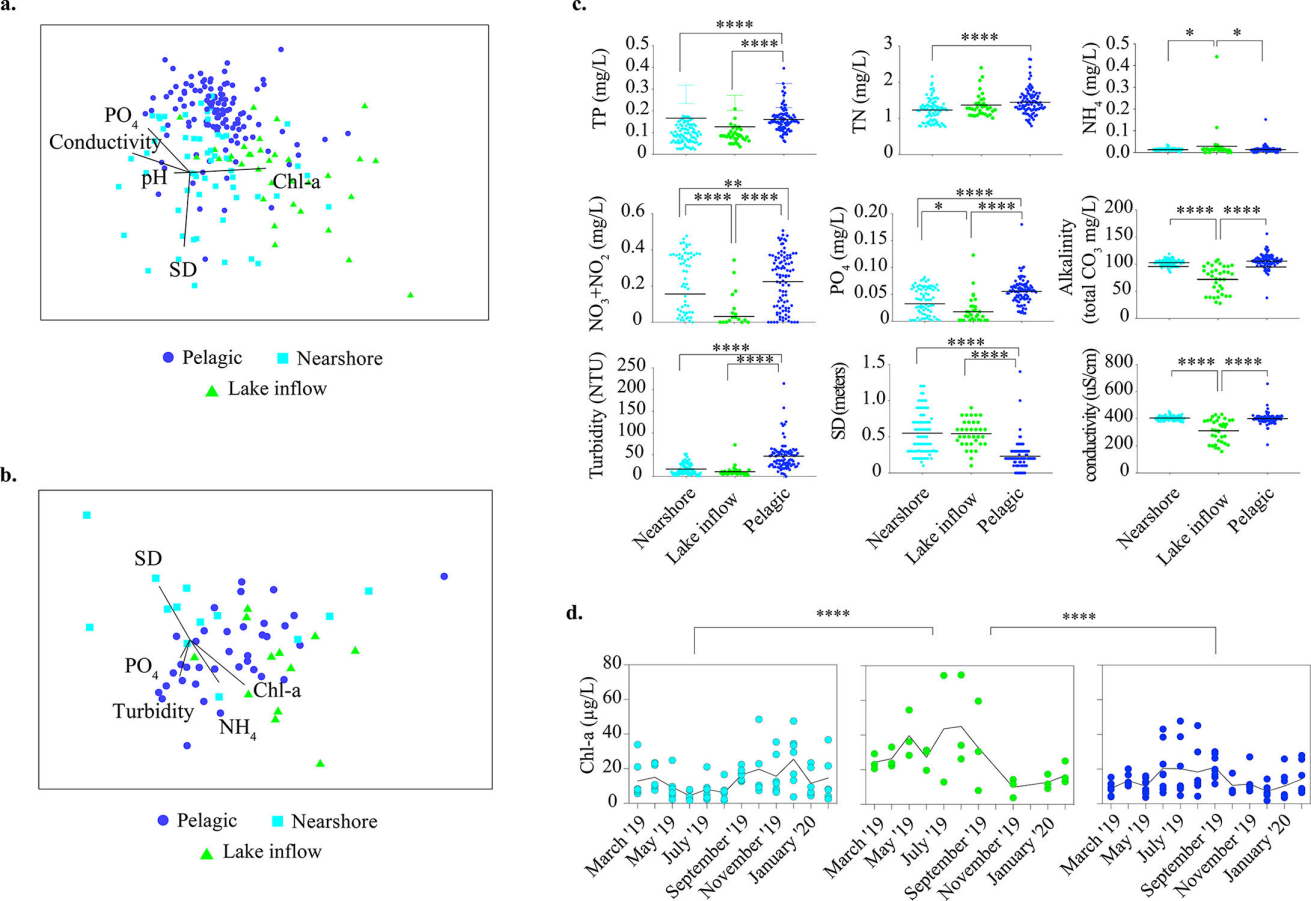
Microbial community dynamics and environmental conditions in Lake O. (a) Non-metric multidimensional scaling (NMDS) analysis (2D stress: 0.17) depicting ecological zones as biogeographical patterns in microbial composition using Bray–Curtis similarities after square root transformation of relative abundances of ASVs from all samples collected from March 2019 to February 2020 from Lake O. Biological environmental stepwise (BEST) (41) analysis was used to determine the environmental variables that significantly correlated (Pearson *R*^2^ = 0.511, *P* = 0.001) to the patterns in microbial composition between samples. (b) NMDS (2D stress: 0.16) analysis depicting ecological zones as biogeographical patterns in functional composition from April 2019 to September 2019. Bray–Curtis similarities were generated for this analysis after square root transformation of copies per million (CPM) bioprocesses defined by Gene Ontology (GO) (43). BEST (41) analysis was used to determine the environmental variables that significantly correlated. (c) A comparison of the physical and chemical factors that corresponded to sample collection for microbial community analyses that differed between ecological zones. Black line represents the average. Pairwise comparisons were performed using the Kruskal–Wallis test and corrected for multiple comparisons using Dunn’s test (*P ≤ 0.05, **P ≤ 0.01, ***P ≤ 0.001, and ****P ≤ 0.0001). (d) Chlorophyll *a* concentration over time in ecological zones. Black line represents the average. Pairwise comparisons of ecological zones were performed using the Kruskal–Wallis test and corrected for multiple comparisons using Dunn’s test (****P ≤ 0.0001). TP = total phosphorus, TN = total nitrogen, SD = Secchi depth.

The microbial community composition of Lake O was evaluated by sequencing 16S rRNA genes in 237 samples collected monthly for 1 year at monitored sites, located at the headwater of the Caloosahatchee River (CR, S77) and downstream on the CRE (S79) and SLRE (S80; Fig. 1; Table S1). The relative abundance of 7,735 amplicon sequence variants (ASVs), representing unique taxa, revealed biogeographical patterns in the lake (Fig. 2a) that were distinct from CR, CRE, and SLRE (41) (analysis of similarity [ANOSIM]; *R* = 0.438, *P* = 0.001; Table S2). The differences detected in microbial community composition reflected previously described ecological zones, i.e., the pelagic and nearshore zones (42). Sites located closest to rivers and canals that deliver the greatest inflows to the lake could be distinguished from these zones (Table S2), hence defined as “lake inflow zone.” Differences observed in microbial community composition based on month (ANOSIM; *R* = 0.258, *P* = 0.001), season (ANOSIM; *R* = 0.128, *P* = 0.001), and site (ANOSIM; *R* = 0.280, *P* = 0.001) were significant, but not as strong as spatial patterns of the zones. The strongest differences in microbial community composition were observed when considering the interaction between zone and month (ANOSIM; *R* = 0.532, *P* = 0.001). Patterns in microbial community composition correlated to chlorophyll *a* (chl*-*a), phosphate (PO_4_), pH, conductivity, and Secchi depth (SD) (41; Fig. 2a; BEST analysis; *R*^2^ = 0.511, *P* = 0.001).

Microbial community functional potential was examined using metagenomes generated from samples collected monthly between April 2019 and September 2019 in Lake O (Table S3; Data S1). Microbial community functional composition was more distinct in the ecological zones (Fig. 2b), which included the lake inflow zone (ANOSIM; *R* = 0.318, *P*, 0.001), when compared between sites (ANOSIM; *R* = 0.290, *P* = 0.001), month (ANOSIM; *R* = 0.079, *P* = 0.041), and season (ANOSIM; *R* = −0.096, *P* = 0.79). Chl-a, ammonium (NH_4_), SD, turbidity, and PO_4_ correlated to patterns in microbial community functional composition (Fig. 2b).

Accordingly, differences in microbial community composition and functional composition between zones corresponded to distinct chemical and physical characteristics (Fig. 2c). Lower nitrate (NO_3_) + nitrite (NO_2_), PO_4_, alkalinity, and conductivity were observed in the lake inflow zone compared to the other zones. The pelagic zone was more turbid yielding lower SD measurements, indicating reduced water clarity likely from sediment resuspension, coinciding with the highest total nitrogen (TN), total phosphorus (TP), NO_3_, and PO_4_. The nearshore zone, with turbidity and SD values comparable to the lake inflow zone, indicated increased water clarity compared to the pelagic zone. The lake inflow zone also had higher chl-a concentration*,* which is a proxy for photosynthetic microorganism biomass, compared to other zones indicating a “hotspot” for blooms (Fig. 2d). The chl-a and cyanobacteria cell concentrations in the lake inflow zone suggested moderate to severe cyanoHABs (5–>50 µg/L chl-a, 10,000–>100,000 cells/mL; 44) from May to September (45; Fig. 2d; Fig. S1). Highest chl-a concentrations were observed in the pelagic zone during this time as well, whereas they were highest in the nearshore zone the following months. Phytoplankton cell counts supported the conclusion that the blooms were dominated by cyanobacteria (Fig. S1).

**Fig 3:**
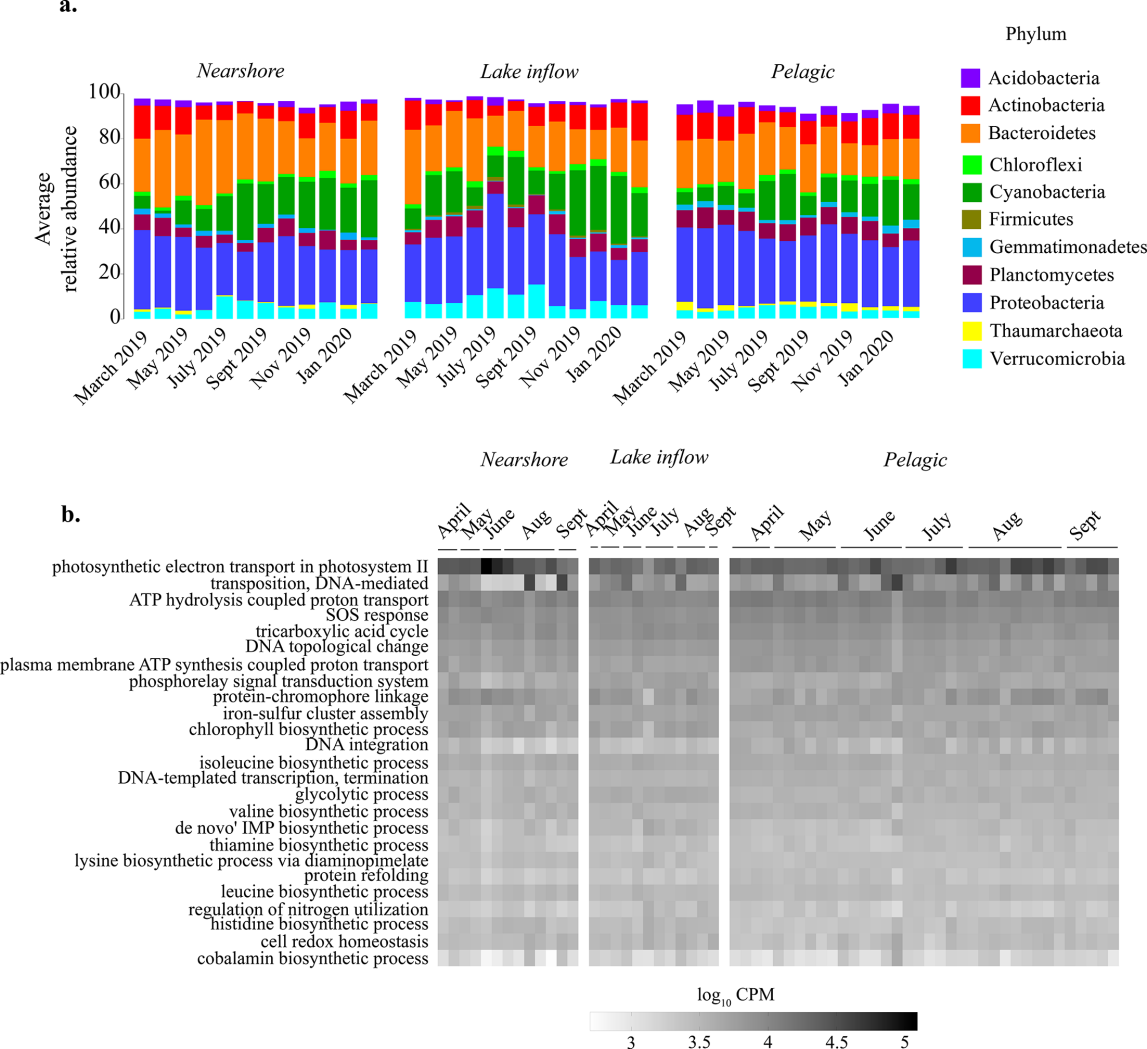
Microbial community composition and functional potential in Lake O. (a) The relative abundance of the top 10 phyla averaged for each month from March 2019 to February 2020 in each ecological zone. The remaining phyla in lower abundances are not shown. (b) The topmost abundant functional pathways shown as the log-transformed CPM of bioprocesses defined by Gene Ontology (43) across ecological zones.

Dominant phyla in all ecological zones were Cyanobacteria, Proteobacteria, Actinobacteria, Planctomycetes, Bacteroidetes, Verrucomicrobia, Chloroflexi, Gemmatimonadetes, Acidobacteria, and Thaumarchaeota, composing more than 95% of the microbial community (Fig. 3a). Differences in microbial community composition between zones were distinguished by the relative abundance of cyanobacteria (SIMPER) (41), with the highest abundance noted for the lake inflow zone. The most abundant functional pathways were involved in photosynthesis, C cycling, pigment production, energy generation, DNA replication and modification, and amino acid and vitamin biosynthesis (Fig. 3b). Differences in functional composition between zones were distinguished by several bioprocesses representing dominant functions of the lake, with the top drivers being photosynthetic electron transport in photosystem II and transposition (SIMPER).

### The microbial community composition and function associated with blooms in Lake O

**Fig 4:**
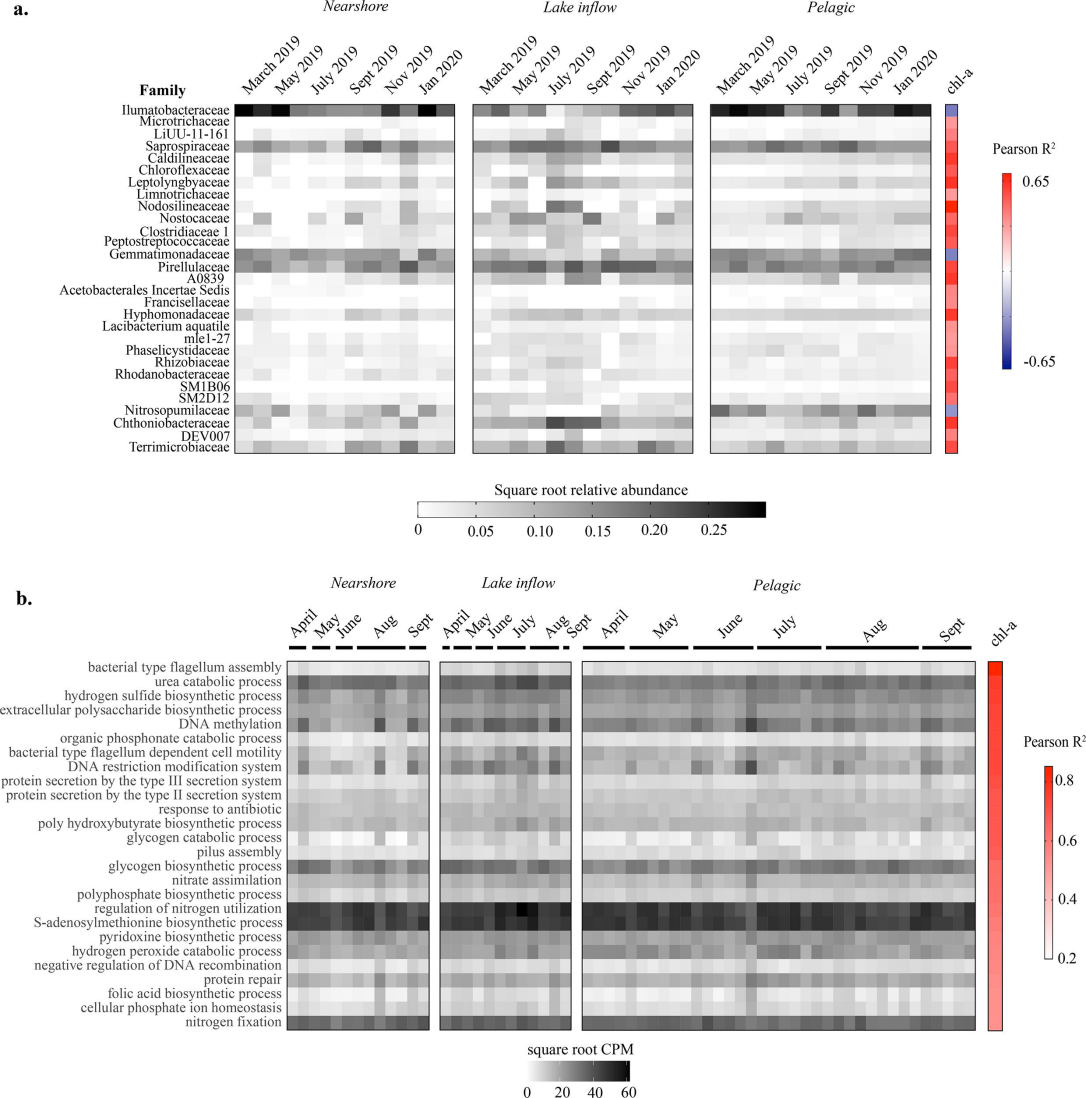
Microbial community composition and functional potential associated with blooms. (a) The families identified in the 16S rRNA data collected from March 2019 to February 2020 that significantly correlated (Pearson *R*^2^ > 0.3 or <−0.3, *P* value <0.05) to chl-a concentration. (b) Pathways identified in metagenomes generated from samples collected from April 2019 to September 2019 classified by Gene Ontology bioprocesses that positively correlated with chl-a concentrations (Pearson *R*^2^, *P* < 0.05); a CPM of >3 in all samples; encoded processes for nutrient utilization, phage defense, and stress response; and supported microbial interactions.

Cyanobacterial families that positively correlated with chl-a included Nodosilineaceae, Leptolyngbyaceae, Limnotrichaceae, and Nostocaceae (Fig. 4a) and non-cyanobacterial families Proteobacteria (*R*^2^ ≥ 0.5), namely, families A0839, Hyphomonadaceae, and Rhizobiaceae. The Chthoniobacteraceae (Verrucomicrobia), Caldilineaceae (Chloroflexi), Clostridiaceae (Firmicutes), Pirellulaceae (Planctomycetes), and Terrimicrobiaceae (Verrucomicrobia) were also found to be strongly correlated with chl-a. Negative correlations to chl-a were moderate and included Illumatobacteraceae (Actinobacteria), Gemmatimonadaceae (Gemmatimondetes), and Nitrosopumilaceae (Thaumarchaeota, Fig. 4a).

Many functional pathways that positively correlated with chl-a were likely reflective of increased biomass expected with blooms, including chl-a biosynthesis, peptidoglycan biosynthesis, and others involved in energy, C, amino acid, and nucleotide metabolism (Data S1). Others could be indicative of differences in life strategies within the microbial community, including those that encode genes for meeting nutrient requirements, such as inorganic and organic N and P metabolism; storage of C as glycogen and polyhydroxybutyrate; storage of P as polyphosphates; vitamin biosynthesis, such as folic acid and pyridoxine; and S cycling (Fig. 4b). Other functions correlating to chl-a were pathways for flagellum and pili assembly; exopolysaccharide biosynthesis; Type III and IV secretion systems; machinery for phage defense, including DNA modification systems and DNA methylation; hydrogen peroxide catabolism; protein repair; and response to antibiotics (Fig. 4b).

### Cyanobacterial diversity in Lake O

A total of 200 cyanobacterial ASVs were detected (Fig. S2a), most of which (120 of the 200) were assigned to the genus *Cyanobium* but may also represent *Synechococcus* or *Vulcanococcus* due to there being a poor resolution within the family Cyanobiaceae at the genus level. On average, these genera were the highest in relative abundance across all zones (Fig. S2b), along with *Microcystis*, *Nodosilinea*, *Cupsidothrix, Cylindrospermopsis, Pseudanabaena, Limnolyngbya, Dolichospermum,* and *Synechocystis* (Fig. S2a and c).

**Fig 5:**
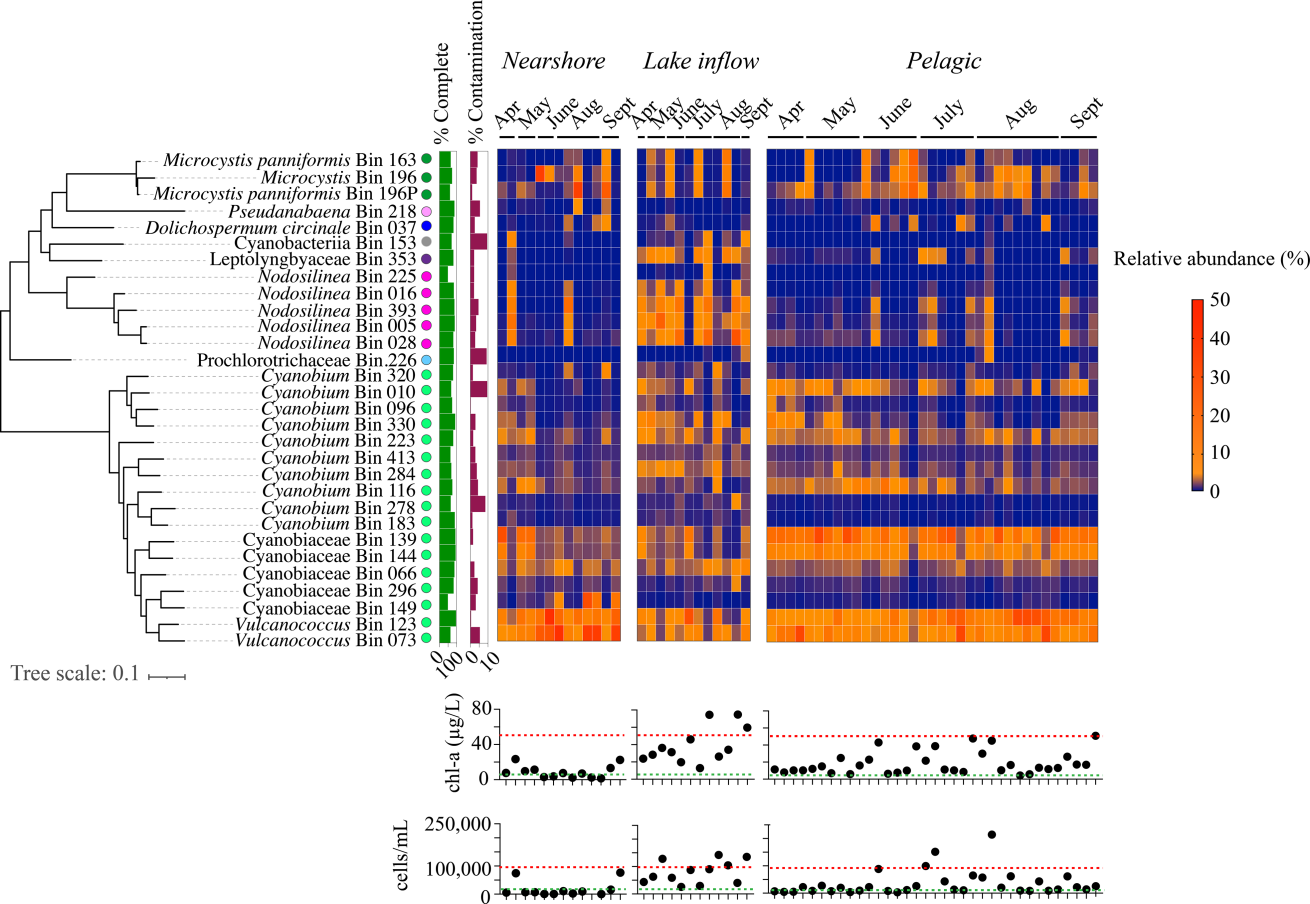
Spatiotemporal trends in the relative abundances of cyanobacteria from samples capturing cyanobacterial bloom season (April–September 2019). MAGs are ordered based on phylogenetic placement using maximum likelihood phylogeny. Taxonomic classification associated with each MAG was determined from GTDB-Tk (46), and different colored circles represent different families of cyanobacteria. Chlorophyll *a* and cyanobacterial cell concentrations (cells/mL) for each sample are shown in the bottom panel. Any value below the green lines represents little to no bloom conditions. Any value above the green lines indicates the threshold for moderate blooms, and the red line and above indicate the threshold for severe bloom conditions (44).

Thirty non-redundant (<99% average nucleotide identity) metagenome-assembled genomes (MAGs), >50% complete and <10% contamination, represented most of the cyanobacterial groups detected in the 16S rRNA sequencing (Fig. 5; Fig. S2a; Table S4). As concluded from the 16S rRNA gene data, the most diverse cyanobacterial MAGs were the family Cyanobiaceae (Fig. 5). Two were identified as *Vulcanococcus* sp. and 10 as *Cyanobium* spp., which were highest in relative abundance in the nearshore and pelagic zones (Fig. 5).

Three MAGs included one *Microcystis* sp. and two *Microcystis panniformis. Microcystis* dominated or co-dominated with other cyanobacteria when the chl-a concentration indicated moderate blooms (5–50 μg/L) in the lake inflow and pelagic zones (Fig. 5; Data S2). The complete microcystin gene cassette was found in *M. panniformis* Bin196P, and a partial cassette was found in *Microcystis* sp. Bin196, indicating these were microcystin producers. Microcystins were the cyanotoxins detected in the highest quantities in Lake O in 2019 (Fig. S3).

Five MAGs annotated as *Nodosilinea* sp. formed a cyanobacteria clade highest in relative abundance in samples from the lake inflow zone of several blooms based on chl-a (>50 µg/L, Fig. 5). Leptolyngbyaceae species, closely related to this group, were also detected. Additional MAGs included *Dolichospermum circinale, Pseudanabaena* sp*.,* and Prochlorotrichaceae in low relative abundance. No microcystin, saxitoxin, anatoxin, or cylindrospermopsin coding genes were detected in *Nodosilinea, Pseudanabaena,* Leptolyngbyaceae*, Dolichospermum, or* Prochlorotrichaceae. Intracellular cylindrospermopsins, anatoxins, and saxitoxins were detected (Fig. S3), but not the genes coding for their production.

### Functional diversity of the Lake O cyanobacterial community

**Fig 6:**
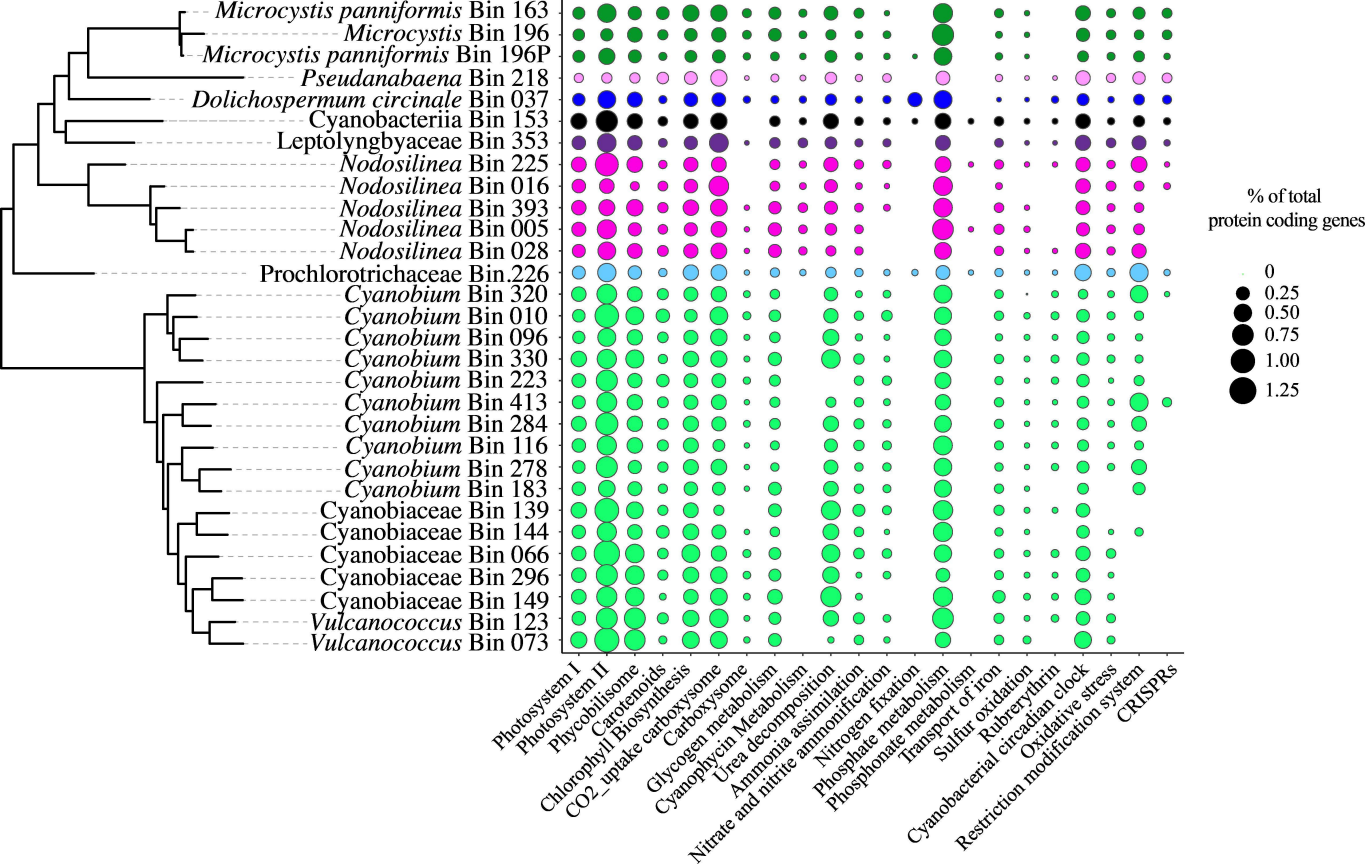
The functional potential of the cyanobacterial community in Lake O. The proportion of protein coding genes in functional pathways relative to the total number of protein coding genes in each cyanobacterial MAG. MAGs are ordered based on phylogenetic placement using maximum likelihood phylogeny, and the colors represent the family to which they belong.

Cyanobacterial MAGs shared functional pathways, including photosynthesis, CO_2_ uptake, pigment production, and NH_4_, NO_3_, and PO_4_ metabolism (Fig. 6; Data S3). All cyanobacterial MAGs contained genes encoding urease and glycogen metabolism (Fig. 6).

**Fig 7:**
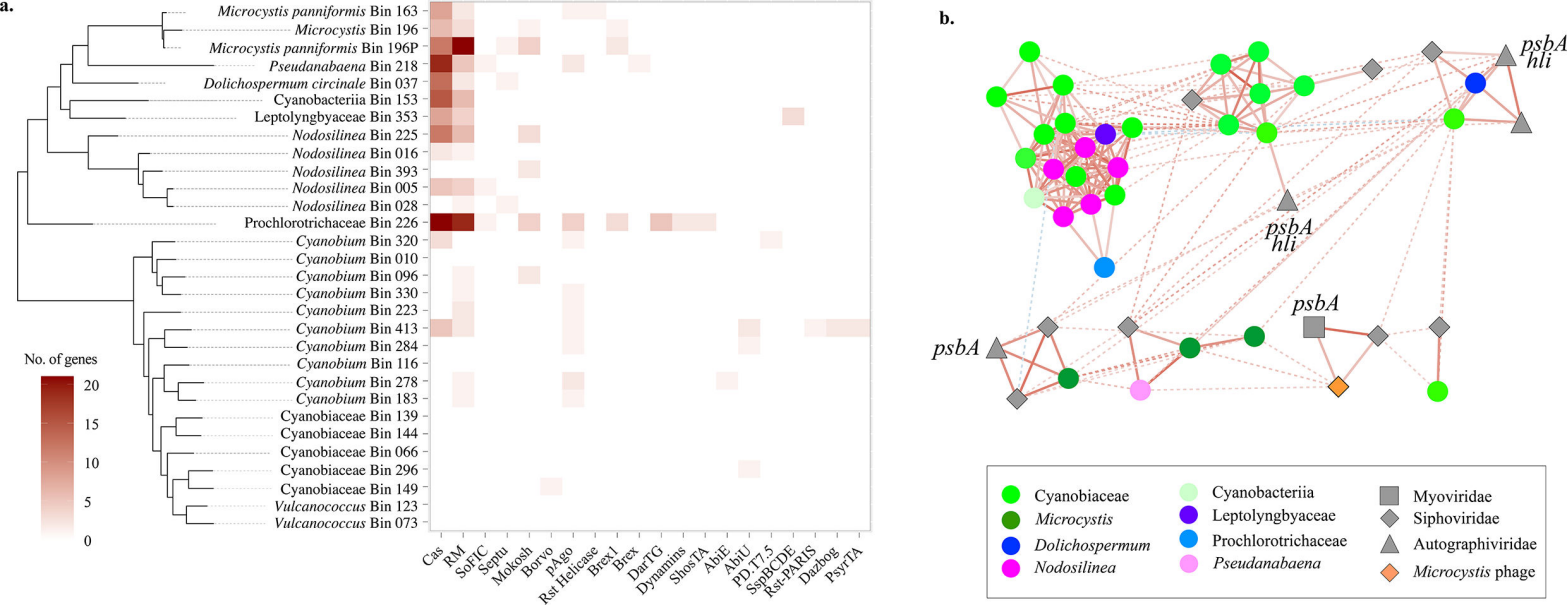
Phage defense systems and phage–cyanobacterial dynamics in Lake O. (a) The distribution and number of phage defense systems in the cyanobacteria of Lake O. (b) Co-occurrence networks based on correlative relationships between cyanophage and cyanobacteria using relative abundances of each genome. Red and blue edges connecting nodes represent positive (Pearson *R*^2^ > 0.3) and negative (Pearson *R*^2^ < −0.3) correlations, respectively. The nodes connected by thicker lines represent the MCL clusters determined. Photosynthesis genes found in a specific cyanophage are indicated: *psbA* encodes a core protein in photosystem II; *hli* = high-light inducible genes.

Genes encoding for cyanophycin metabolism were detected in *Microcystis, Dolichospermum, Pseudanabaena, Nodosilinea*, Leptolyngbyaceae, and Prochlorotrichaceae (Fig. 6), but not in *Vulcanococcus, Cyanobium*, and other Cyanobiaceae MAGs. Classification of phage defense systems with Rapid Annotation for microbial genomes using Subsystems Technology (RAST) (47) and Defense Finder (48) indicated that phage defense systems were diverse across Lake O cyanobacteria. Most cyanobacteria encoding them had more than one, but they were differentially present across cyanobacterial genomes (Fig. 7a). CRISPR genes were present in only two *Cyanobium* but detected in *Microcystis, D. circinale, Pseudanabaena*, Leptolyngbyaceae, Prochlorotrichaceae, and three *Nodosilinea* genomes. While results for the presence of RMs differed between the two methods, fewer genes for RMs were observed in the picocyanobacteria (*Cyanobium, Vulcanococcus*, and other Cyanobiaceae) compared to bloom-forming cyanobacteria (Fig. 6 and 7a). The differences in the presence of CRISPRs and RMs were confirmed to be not related to missing sequence information or binning issues by analyzing an additional 369 publicly available genomes of previously sequenced and cultured cyanobacteria representing cyanobacterial families observed to be present in Lake O (Fig. S4). Most *Cyanobium* also had genes for phage argonautes (pAgos), as did *Microcystis* sp*.* Bin163 and *Pseudanabaena*.

### Characterization of Lake O cyanophages

Thirteen non-redundant (<99% average nucleotide identity) genomes were identified as families of Caudoviricetes (Myoviridae, Autographiviridae, and Siphoviridae) ranging from ~40 to 190 kbp (Fig. S5; Data S4). All were considered likely lytic phages since their genomes were not flanked by host DNA. Marker gene analysis and results from vConTACT2 suggested their hosts were picocyanobacteria*,* specifically *Synechococcus* (Fig. S5). Their genomes carried genes for DNA and RNA replication and modification, transfer RNAs, and nucleotide metabolism, including auxiliary metabolic genes, such as thymidylate synthase (*thyX*) and ribonucleotide reductase (49; Table S5). Some phage genomes from the families Autographiviridae and Myoviridae contained *psbA*, encoding a core protein in photosystem II, the most abundant pathway detected in Lake O. The phage genome in the Myoviridae family also contained ferredoxin, whereas phages from the Autographiviridae family contained high-light-inducible (*hli*) proteins. Additionally, the phage genome from Myoviridae family had *phoH*, a part of the Pho regulon for PO_4_ metabolism (50).

Several *Cyanobium, Vulcanococcus,* and Cyanobiaceae co-occurred with Autographiviridae genomes with *psbA* and high-light-inducible proteins (Fig. 7b). These MAGs also co-occurred with Siphoviridae genomes. Several cyanophages co-occurred with *Microcystis* and *Pseudanabaena* or *Dolichospermum* (Fig. 7b; Data S4). One Autographiviridae with *psbA* co-occurred with *Microcystis*. No cyanophage genomes co-occurred with *Nodosilinea*, Leptolyngbyaceae, or Prochlorotrichaceae MAGs (Fig. 7b).

## DISCUSSION

Management practices and research on cyanoHABs have focused on controlling nutrient input, but other factors are involved in constraining cyanoHAB development and duration. The physical and chemical environments have had attention, and the biology of lakes experiencing cyanoHABs should also be considered. Microbial processes are key in biogeochemical cycling and ecosystem function, and studying blooms as a microbial ecosystem can help identify indicators for the processes and transformations that are important in bloom success (51). In this study, the microbial ecology of Lake O was analyzed in conjunction with the physical and chemical environmental parameters to identify holistically the drivers of cyanoHABs.

### Microbial community dynamics reflect ecological zones and bloom hotspots in Lake O

Spatiotemporal resolution of the sampling carried out in this study offered a range of physical and chemical conditions influencing microbial community composition and functional potential. Both reflected biogeographical patterns representing ecological zones in Lake O, originally defined by physical, chemical, and macro-biological features (42, 52). Physical and chemical characteristics analyzed in this study from March 2019 to February 2020 were aligned with previous descriptions, namely, the pelagic zone having reduced water clarity and high nutrient concentration and the nearshore zone with high water clarity and lower nutrient concentration. Additionally, the microbial community dynamics were distinct at sites near major inflows into Lake O, defined as the lake inflow zone. The lake inflow zone included sites near the mouths of the Kissimmee River (KissR0.0), Indian Prairie Canal (Polesout), and Taylor Creek (LZ2) in the northwest part of the lake, which delivers the largest inflows into the lake that contains nutrient-rich water impacted by agriculture and animal farming (53, 54). Corresponding to differences in microbial community composition and functional potential, the chemistry at these sites was characteristic, namely, higher chl-a concentration, suggesting that lake inflow areas are “hotspots” for blooms. Rivers and canals flowing into Lake O are known to carry high nutrient loads. A low concentration of nutrients at the sites may result from increased cyanobacterial activity, where a drawdown of dissolved nutrients may be expected.

### Microorganisms and functional potential associated with blooms

Both microbial community composition and functional potential were found to be correlated with chl-a*,* suggesting biological activities associated with the blooms. The extent of sampling provided a wide range of chl-a concentrations that represented a scale of bloom severity to determine which patterns were associated with blooms. Results of several studies have shown that microbial communities change according to cyanobacterial abundances and stage of bloom (14, 16, 17) and that changes can differ across an ecosystem (25). In Lake O, several non-cyanobacterial families were found to increase in relative abundance with increasing bloom severity. These were members of phyla that were previously detected during blooms and tightly associated with colonies (12, 25). Firmicutes and Proteobacteria have been shown to induce colony formation by *Microcystis*, promoting bacterial interactions (17, 19). Actinobacteria have been observed in high abundance in lakes where there were no blooms (55) and are more likely to be free-living rather than associated with *Microcystis* colonies (17). In Lake O, some negative associations with increased bloom severity were noted with Actinobacteria.

While changes in the composition of a microbial community associated with blooms, cyanobacterial aggregates (12, 25, 56), and genus of bloom former (17, 57) have been observed, microbial community functions during a bloom appear to be more conserved (17, 56). Functional pathways found to be correlated to chl-a in Lake O aligned with similar observations for other lakes. Bloom severity was found to be associated with genes encoding motility, biofilm formation, and exchange of metabolites and genetic material, indicating that microbial activities, such as chemotaxis, genome rearrangement, and horizontal gene transfer occur during blooms (18, 34, 58, 59). Several studies provide evidence of microbial communities being present during blooms or colonizing cyanobacterial aggregates that are also involved in N, P, C, and S cycling, fatty acid metabolism, and vitamin biosynthesis (10, 18, 20, 21, 25), which aligns with the results of the study reported here. It has been demonstrated that the catalase activity of heterotrophic bacteria support the fitness of strains of *Microcystis* by reducing oxidative stress (22). This is a mechanism possibly important for cyanoHAB resilience in Lake O, where increased hydrogen peroxide activity and pyridoxine biosynthetic processes were linked to bloom severity. Enrichment of antimicrobial resistance (AMR) genes has been associated with cyanoHABs (60, 61), which contributes to the dissemination of AMR genes in the environment. Indeed, urban and agriculture runoff is known to disseminate AMR genes in aquatic ecosystems in addition to being a source of nutrients (62, 63). Along with the increase in genes involved in responses to possible biological stressors, like phage infections, the functions associated with bloom severity in Lake O suggested that the microbial communities associated with blooms are more adaptable to dynamic environments. In general, this seems to be a quality of microbial communities across Lake O; transposition, which could promote adaptive evolution of these communities, and SOS response were two highly abundant functional pathways in all samples.

The enrichment of genes encoding for nutrient storage with increasing chl-a also supports that the microbial communities are more resilient in blooms. The production of molecules to store C and P can allow bacteria to persist in environments with fluctuating nutrient supplies (64, 65) and during times of nutrient limitation, providing an important message with respect to monitoring of relationships between nutrients and blooms. That is, more biomass may not always correspond to more nutrients, and reducing nutrients may not always correspond to reduced biomass, at least over short timescales (66). This physiological aspect of potential nutrient storage associated with blooms should be considered in modeling and forecasting cyanoHABs.

### The cyanobacteria of Lake O are taxonomically and functionally diverse

Cyanobacteria present in Lake O were identified and characterized using molecular tools revealing the diversity of picocyanobacteria, *Microcystis* species, and other potential bloom formers in Lake O. In the past several years, blooms of *Microcystis* reported in Lake O have received the most attention due to their negative impact on the CRE and SLRE, which receive outflows from Lake O (33, 35, 37). The *Microcystis* species found to dominate in the lake during sampling was *M. panniformis*. Interestingly, while *Microcystis* was present and presumed to contribute to the moderate blooms in the lake inflow and pelagic zones, dominant bloom formers in Lake O in our sampling were the filamentous cyanobacteria. MAGs representing *Nodosilinea* sp. were of the highest relative abundance during more severe blooms. Blooms caused by *Nodosilinea* have not been reported to occur on Lake O but were reported elsewhere (67). The Leptolyngbyaceae family, to which *Nodosilinea* belongs, is known to be polyphyletic and has a taxonomy difficult to resolve. Historically, other filamentous taxa have been reported to be part of blooms in Lake O, namely, *Lyngbya* (68). Thus, such blooms have been overlooked or possibly represent a novel bloom community in Lake O, containing genotypes of filamentous non-N_2_ fixing cyanobacteria.

MAGs representing picocyanobacteria in Lake O were highly diverse and in high relative abundances when blooms were least severe, notably in the nearshore zone, aligning with an assumption that picocyanobacteria are not associated with blooms in lakes (69). High relative abundance of active picocyanobacteria has been reported to occur in other eutrophic lakes, and they are recognized as important contributors to ecosystem function (70). However, they are not usually considered in the analyses of bloom ecology, despite their importance in biogeochemical cycles in these ecosystems and blooms in coastal marine ecosystems (71). Some studies suggest that picocyanobacteria can outcompete bloom-forming cyanobacteria under certain conditions because of their faster growth rates and lower nutrient requirements (72–75). The co-occurrence of picocyanobacteria and bloom-forming cyanobacteria, such as *Nodosilinea*, Prochlorotrichaceae, Leptolynbyaceae, and *Dolichospermum*, in this study, indicated that they do at times co-exist and likely compete for resources. Thus, understanding how they co-exist or are outcompeted by bloom-forming cyanobacteria may shed light on cyanoHAB ecology and warrants further investigation.

Storage of N via cyanophycin was a distinctive trait missing from Lake O picocyanobacteria. Cyanophycin is postulated to provide a competitive advantage to those cyanobacteria that bloom, such as *Microcystis*. However, this has not been confirmed experimentally. In studies of a *Synechocystis* sp., cyanophycin provided wild-type cells an advantage over a cyanophycin mutant during fluctuating N supply and N-limiting conditions (76). In dynamic environments, like Lake O, which are shallow, experience regular mixing, and are frequently N-limited, this is an advantage. Interestingly, *Synechococcus* has been associated with low N in other eutrophic lakes (25) and fast growth rates under low N conditions (75). The lack of cyanophycin may reflect the differences in lifestyle strategy and nutrient requirements between these organisms and highlight what may regulate this relationship.

Differences in phage defense mechanisms of Lake O cyanobacteria were also observed. Bloom-forming cyanobacteria were more enriched in CRISPRs and RMs in general, whereas phage defense mechanisms in picocyanobacteria, if they possessed them at all, were less abundant. Bloom-forming cyanobacteria are known to possess diverse antiphage defense systems, including CRISPRs (77, 78), and the lack of CRISPRs in picocyanobacteria is not a new observation (79). The results from this study support this, while also demonstrating that phage defense systems are generally fewer in picocyanobacteria. This suggested that the ability to defend against phage, specifically through these mechanisms, is an advantage for bloom-forming cyanobacteria (77–79).

Phage defense systems were diverse across the cyanobacterial population in Lake O. Additionally, genes for phage defense systems with different mechanisms were present, including those involved in nucleic acid degradation, such as CRISPR, RMs, phage argonautes (pAgos), bacteriophage exclusion, and *SspBCDE* (48) and abortive infection and the like, such as AbiE, AbiU, and dynamins (Fig. 7) (48, 80). Indeed, some of these systems have dual functionality, such as CRISPRs and pAgos, of which both offer sequence-specific defense (81). Others were previously described as toxin–antitoxin systems, such as ShosTA, PsyrTA, and DarTG, while the mechanisms of many others with confirmed phage defense remain unclear (48, 82, 83). The diversity of phage defense systems is likely a consequence of host–phage co-evolution and may provide increased host resistance to one phage or a wider range of phages and prevent the emergence of resistant phage (84). The important role of cyanophage in bloom ecology has been recognized previously (29, 30). However, these observations highlight the importance of phages as a constraint on co-existing and competing cyanobacteria in Lake O, while also suggesting that the diversity of specific defense systems may be involved in the succession and proliferation of specific cyanobacteria that bloom (79).

### A role for cyanophage that infects picocyanobacteria in blooms

Following the observation of differential phage defense mechanisms in the cyanobacteria of Lake O, several phages that infect picocyanobacteria were recovered. Some phages contained auxiliary metabolic genes that can alter cellular metabolism, specifically some involved in photosynthesis, further highlighting the role of phage in the ecology of Lake O picocyanobacteria. The co-occurrence of phages with *Microcystis, Dolichospermum,* and *Pseudanabaena* suggested infection of picocyanobacteria was correlated with higher abundances of cyanobacteria that bloom. This suggests that infection of picocyanobacteria, whose phage defense mechanisms are fewer and less specific, may positively impact bloom-forming taxa. This dynamic supports the “kill-the-winner” hypothesis (85, 86) in bloom ecology, which has been suggested in previous work (87). In this case, picocyanobacteria would be the best competitor whose abundances are maintained at a certain threshold due to phage infection, and bloom-forming taxa would be the defense specialist who are more equipped to evade phage infection and thus outcompete the picocyanobacteria (85–87). Quantitative evaluation of phage infection and bacterial protection dynamics could shed light on this hypothesis. Phage–host relationships are complex on their own, and the role of CRISPRs and other phage defense in kill-the-winner is unclear (86). However, there is a clear trade-off between having more phage defense systems and competition (86). Thus, these observations offer some insight into the complex interactions, including the on-going battle between phage and cyanobacterial host, in microbial ecosystems influencing the ability of specific cyanobacteria to bloom.

### Conclusions

This study focused on changes in microbial community composition and functional potential related to specific processes and interactions influencing cyanobacterial blooms in addition to the physical and chemical environment alone. Microbial interactions between heterotrophic microorganisms and cyanobacteria, often overlooked in cyanobacterial bloom ecology of Lake O, are important influences on cyanoHABs. Cyanobacterial blooms are best studied as a microbial ecosystem rather than solely a collection of bloom-forming taxa in a lake. Routine quantification of the microbial ecosystem may represent an important component to measure in addition to the physical and chemical parameters of the lake. By including the biology of a cyanobacterial bloom in Lake O, the knowledge of this ecosystem is expanded beyond nutrient analysis and highlights the complexity within a microbial ecosystem. Information about the microbial functions and interactions, as well as the role of phage coould enhance the understanding of the entire system and processes to allow effective prediction, prevention, and mitigation of blooms.

## MATERIALS AND METHODS

### Sample collection and DNA extraction

Surface water dip samples at 0.05 m depth were collected monthly by the South Florida Water Management District (SFWMD) using a decontaminated bottle (washed with hot water and Liquinox soap, rinsed with tap water three times, rinsed with 10% (wt/wt) hydrochloric acid, and rinsed with analyte free water three times) at 19 sites in Lake O from March 2019 to February 2020 (Fig. 1; Table S1). The U.S. Geological Survey in Orlando, FL, collected surface water at sites S-79 on the CRE and S-80 on the SLRE. Water samples were stored on ice and shipped overnight to the USGS Caribbean–Florida Water Science Center where the water samples were filtered in 0.22 µm Sterivex cartridges (Millipore, SVGP01050), frozen and stored at −20°C, and transported on ice to Nova Southeastern University in Dania Beach, FL. The Sterivex cartridges were carefully opened using a pipe cutter, and the filter was removed. DNA was extracted using the DNeasy PowerLyzer PowerSoil DNA extraction kit (Qiagen, 12855-100) following the manufacturer’s protocol. Blank “reagent-only” extractions were performed. All environmental data including chl-a (µg/L), SD (cm), total suspended solids (mg/L), turbidity (NTU), alkalinity (as total CaCO3), NH_4_ (mg/L), color (PTU), depth (m), DO (mg/L), NO_3_ (mg/L), NO_3_ + NO_2_ (mg/L), pH, PO_4_ (mg/L), conductivity, temperature (°Celsius), TN (mg/L), TP (mg/L), and volatile suspended solids (mg/L) were downloaded from the DBHYDRO SFWMD environmental database that stores historic and current hydrologic, meteorologic, hydrogeologic, and water quality data (https://www.sfwmd.gov/science-data/dbhydro) collected at the same date and time of the sample collection for DNA analyses.

### 16S rRNA amplicon sequencing and analysis

Purified DNA was amplified following the 16S Illumina amplicon protocol recommended by the Earth Microbiome Project (88) as previously used (89, 90). Briefly, barcoded primers 515F and 806R (88) were used to amplify the V4 region of the 16S rRNA gene (https://earthmicrobiome.org/protocols-and-standards/16s/). Initial bioinformatic analysis was performed within the Qiime2 (version 2019.10) environment (91). Forward and reverse reads were paired and demultiplexed, and dada2 was used to trim forward and reverse reads based on their quality profiles, merge paired reads, filter forward and reverse reads based on total read quality (*q*-score >29), and check for chimeras (“consensus”). The identification of taxa was assigned to ASVs using the silva classifier (silva-132-99-515-806-nb-classifier). Mitochondrial and chloroplast ASVs and ASVs in sample extraction reagent blanks were removed for downstream analysis. Relative abundances of ASVs were determined in R (92) using the vegan package (93) function decostand after the removal of singletons, doubletons, and ASVs that were represented in less than 0.01% of samples using colSums.

Multivariate statistical analyses using the relative abundances of ASVs were performed in Primer-E (v7) (41), including NMDS and ANOSIM for identifying dissimilarities between groups, SIMPER for identifying what variables contribute to the greatest differences between groups, and BEST, which identifies environmental factors that correlate to microbial community and functional composition (41). Nearshore and pelagic sites were assigned a zone based on previous literature (94). Since zones do not have geographically defined borders, sites near the transition of the pelagic and nearshore zones were assigned based on which zone they were more similar to using ANOSIM results.

Relative abundances of families and chl-a concentrations were correlated (Pearson *R*^2^) in R (92) using the package “Hmisc (95)” and the command “rcorr.” The heatmap displaying families that correlated to chl-a was created using ggplot2 (96) package in R (92) using relative abundances of each family square root transformed for visualization purposes.

### Metagenomic sequencing and bioinformatic processing

Seventy-one DNA samples from Lake O were chosen for metagenomic sequencing (Table S3). DNA was sequenced using the Illumina HiSeqX with 150 bp paired-end reads. Raw reads were uploaded for taxonomic annotation on the CosmosID-HUB using a patented and benchmarked kmer-based algorithm against the database GenBook (https://docs.cosmosid.com/docs/metagenomic-classification). For additional analyses, sequencing reads were filtered and trimmed for quality and adapter removal using bbduk (97) (min = 25, qtrim = rl, trimq = 20) and then co-assembled by location using MEGAHIT (98) (kmer = 55, 77, 99; Table S3). To preserve genome variability across the lake but reduce redundancy in recovered MAGs, co-assemblies by site were performed due to the strong spatial trends observed in the microbial community composition elucidated in the 16S rRNA gene sequencing data.

### Metagenome functional analysis

Reads were functionally annotated (https://docs.cosmosid.com/docs/methods#functional-classification-methods) in the CosmosID-HUB Microbiome Platform as previously described (99–101). Briefly, reads passing quality checks were searched against the protein sequence database, UniRef90 (102), to assign gene families using HUMAnN2. Reads were mapped to representative gene sequences and weighed by mapping quality, coverage, and gene sequence length to estimate community-wide weighted gene family abundances (103). Detailed information on mapping quality and coverage can be found in Franzosa et al. (103). Gene families were then regrouped to establish GO terms and determine their abundances. The abundance values were normalized using total-sum scaling (104) normalization to produce CPM units.

CPM of the most abundant GO term representing biological processes (CPM > 3 across all samples) and chl-a concentrations were correlated (Pearson *R*^2^) in R (92) using the package “Hmisc (95)” and the command “rcorr.” The heatmap to display functions involved in nutrient cycling, biological defense, and stress response was created using the ggplot2 (96) package in R (92) using copies per million of each pathway and square root transformed for visualization purposes.

### MAG recovery and analysis

Contig binning for each co-assembly, quality checks, and annotation was performed using apps within the Kbase platform (105). MetaBAT2 (106) and Maxbin2 (107) were used to assign contigs from each assembly to bins, and these bins were optimized using DAS Tool (108). Bins were filtered for quality with CheckM (109). Cyanobacterial MAGs (>50% complete with <10% contamination) were dereplicated at 99% average nucleotide identity (dRep) (110). GTDB-Tk (46) and the SpeciesTreeBuilder app, which uses FastTree2 (111) to build a maximum likelihood phylogenetic tree, were used for taxonomic classification and phylogenetic placement of dereplicated bins. Prodigal (112) and RAST (47) were used to identify and functionally annotate open reading frames (ORFs). View Functional Profile was used to determine the proportion of protein coding genes in a functional pathway relative to the total number of protein coding genes in each MAG. Reference genomes of cyanobacterial families identified in Lake O came from NCBI directly through KBase (105) and were annotated using RAST-Tk, and the phylogenetic relationships of cyanobacteria with different functions were observed using the SpeciesTreeBuilder app. Cyanobacterial MAGs were searched for phage defense systems using Defense Finder (https://defensefinder.mdmlab.fr/) (48).

Cyanobacterial MAGs and metagenomic assemblies were searched for cyanotoxin genes using a translated blast approach. Nucleotide sequences of the MAGs were searched against a database of protein sequences with verified function encoding McyD for microcystins, SxtA for saxitoxins, CyrK for cylindrospermopsins, and AnaC for anatoxins using BLASTx. Candidate sequences were then screened against the nr database using BLASTx to confirm their homology to the designated protein.

Paired-end reads were competitively mapped to each MAG from each sample using the Bowtie2 (113) v2.32 app in Kbase (105), which outputs alignment statistics, including average coverage. The average coverage of contigs in a MAG was averaged to achieve the average coverage for each MAG. Relative abundances were calculated using the average coverage of each MAG divided by the total number of reads for each sample (Data S2).

### Detection, validation, and analysis of cyanophage

Viral signatures in metagenome co-assemblies were detected in Virsorter1.0.5 (114) in Kbase (105). Contigs in categories 1, 2, 4, and 5 were assessed for completeness and quality using CheckV (85). Contigs that were >90% complete and >10,000 bp and identified as high quality were found only in categories 1 and 2. Putative viral genomes were additionally assessed for host range by manually annotating ORFs with RAST-Tk and aligning all called ORFs to the nr database (115) (as of 29 March 2022) using BLASTp to confirm the contigs were viral and identify terminase, capsid, and ribonucleotide reductase marker genes (116). vConTACT2 (117) was used to confirm host identities from these annotations. Viral genomes were run through Genomad web application at https://nmdc-edge.org/home (118) for viral taxonomy. Additional phylogenetic analysis was performed to achieve higher resolution (118) of taxonomy and support host calls. Newly identified terminase sequences and all reference terminase sequences from UniProt (102) (clustered at 97% identity using CD-HIT) were aligned using MAFFT (119) and trimmed with trimAI (120) (gap threshold = 0.8). A maximum likelihood tree was built to visualize the relationships of the terminase sequences with FastTree2 (117). Phage abundances were determined using the same methods as the MAG abundances. Relative abundances of cyanobacteria and putative cyanophage were correlated (Pearson *R*^2^) in R (92) using the package “Hmisc” (95) and the command “rcorr.” Significant correlations (*R*^2^ > 0.3, *R*^2^ < −0.3, *P* < 0.05) were used for generating MCLusters (121) in co-occurrence networks visualized in Cytoscape (122).

### Phytoplankton counts and toxin measurements

Total anatoxins, cylindrospermopsins, microcystins/nodularins (SAES-ADDA), and saxitoxins were measured by enzyme-linked immunosorbent assay (Eurofins Abraxis, Inc., Warminster, PA, USA) as described in Loftin et al. (123, 124) and Graham et al. (125).

Live samples were sent to PhycoTech, Inc. (St. Joseph, MI; https://www.phycotech.com/) for identification and enumeration of phytoplanktons. An aliquot of 125 mL of the original sample was transferred into a plastic amber bottle for live algal analysis. The bottles were placed in a cooler on ice and shipped overnight to PhycoTech, Inc., where the live samples are run through an Imaging FlowCytobot (McLane Research Labs).

## Data Availability

All 16S rRNA gene amplicon sequencing data, metagenomic data, and metagenome-assembled cyanobacterial and viral genomes are available under the BioProject PRJNA813570 (available at NCBI). Phytoplankton cell concentrations and cyanotoxin concentrations are available elsewhere (45). All environmental data used in this study are publicly available on DBHydro (http://my.sfwmd.gov/dbhydroplsql/show_dbkey_info.main_menu).

## SUPPLEMENTAL MATERIAL

Data S1 msystems.00709-23-s0001.xlsx Gene ontology bioprocesses in Lake Okeechobee.

Data S2 msystems.00709-23-s0002.xlsx Cyanobacteria metagenome assembled genome abundances and environmental metadata associated with metagenome samples.

Data S3 msystems.00709-23-s0003.xlsx Abundances of pathways within each cyanobacterial metagenome assembled genome from Lake Okeechobee.

Data S4 msystems.00709-23-s0004.xlsx Cyanophage genome abundances and information in Lake Okeechobee.

Supplemental Material msystems.00709-23-s0005.pdf Figures S1-S5.

Table S1 msystems.00709-23-s0006.xlsx Information for 16S rRNA amplicon libraries from samples collected in Lake Okeechobee from March 2019 to February 2020.

Table S2 msystems.00709-23-s0007.xlsx ANOSIM comparing dissimilarities in microbial community composition across ecological zones in Lake Okeechobee.

Table S3 msystems.00709-23-s0008.xlsx Summary information for all metagenomes collected in Lake Okeechobee in 2019.

Table S4 msystems.00709-23-s0009.xlsx Cyanobacterial metagenome assembled genome (MAG) statistics and information.

Table S5 msystems.00709-23-s0010.xlsx Auxiliary metabolic genes present in picocyanobacterial cyanophage genomes recovered from metagenomes from Lake Okeechobee in 2019.

ASM does not own the copyrights to Supplemental Material that may be linked to, or accessed through, an article. The authors have granted ASM a non-exclusive, world-wide license to publish the Supplemental Material files. Please contact the corresponding author directly for reuse.
