# Supplementary material for: Microbial diversity, genomics, and phage–host interactions of cyanobacterial harmful algal blooms
Source: mSystems. 2024 Jun 10;9(7):e00709-23. doi: 10.1128/msystems.00709-23 (PMC11265339; doi:10.1128/msystems.00709-23)
Supplement: Supplemental Material — Figures S1-S5. [file msystems.00709-23-s0005.pdf]

SUPPLEMENTAL INFORMATION

Microbial diversity, genomics, and phage-host interactions of cyanobacteria harmful algal blooms

Lauren E. Krausfeldt[1*], Elizaveta Shmakova[1], Hyo Won Lee[1], Viviana Mazzei[2], Keith A. Loftin[3], Robert P. Smith[1,4], Emily Karwacki[2], Eric P. Fortman[1], Barry H. Rosen[5], Hidetoshi Urakawa[5], Manoj Dadlani[6], Rita R. Colwell[7], and Jose V. Lopez[1]

1. Department of Biological Sciences, Guy Harvey Oceanographic Center, Nova Southeastern University, Dania Beach, FL
2. U.S. Geological Survey Caribbean-Florida Water Science Center, Orlando, FL
3. U.S. Geological Survey Kansas Water Science Center, Lawrence, KS
4. Cell Therapy Institute, Kiran Patel College of Allopathic Medicine, Nova Southeastern University, Fort Lauderdale, FL
5. Department of Ecology and Environmental Studies, Florida Gulf Coast University, Fort Myers, FL
6. CosmosID, Rockville, MD
7. Institute for Advanced Computer Studies, University of Maryland College Park, College Park, MD

*Corresponding author

Dr. Lauren E. Krausfeldt, PhD
Guy Harvey Oceanographic Center
Nova Southeastern University
8000 N. Ocean Drive
Dania Beach, FL 33004
lkrausfe@nova.edu

Supplemental Figures

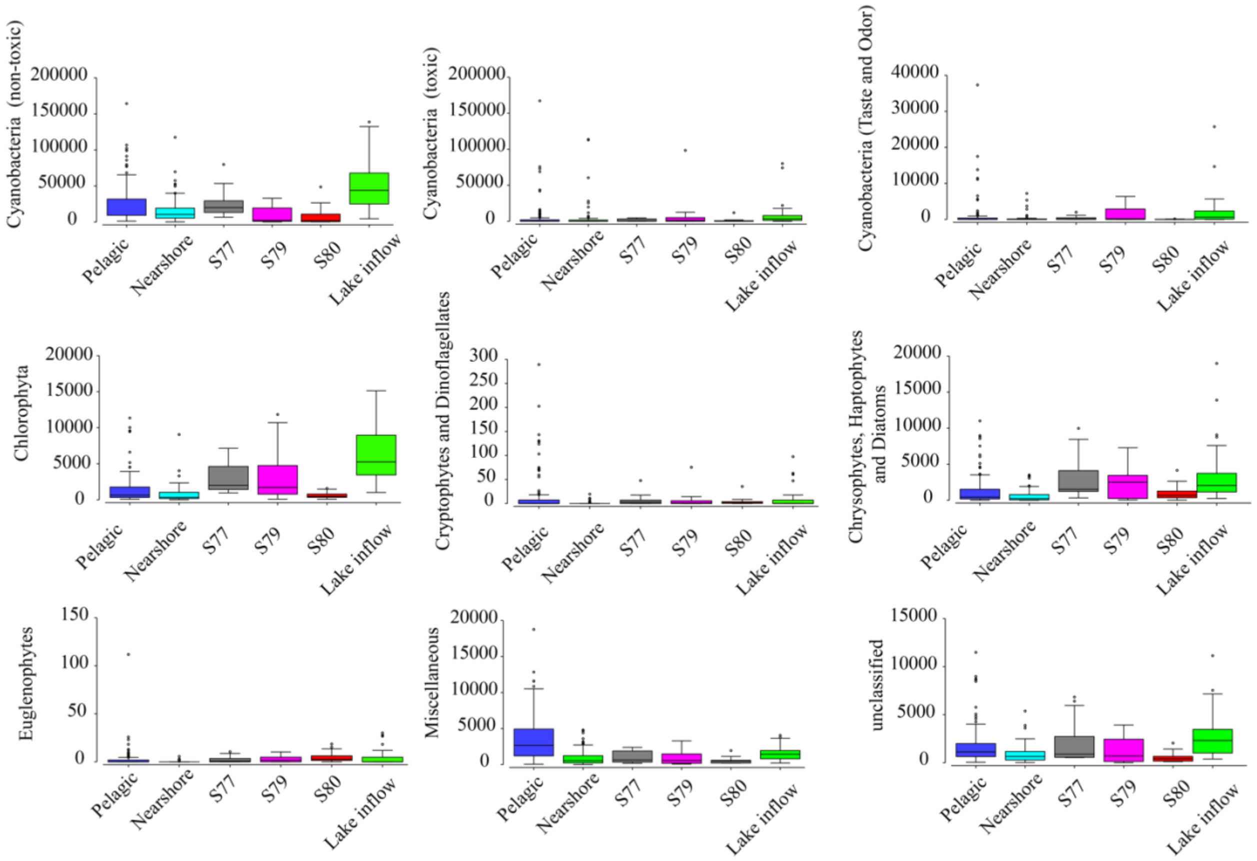

Figure S1. Cell counts (cells/mL) of different phytoplankton functional groups in ecological zones, S77 (located on the upper Caloosahatchee River), S79 (located on the Caloosahatchee River Estuary), and S80 (located on the St. Lucie River Estuary) represented by boxplots. Non-toxic, toxic, and taste and odor groups of cyanobacteria were classified by Phycotech. Toxic cyanobacteria included the genera *Anabaenopsis*, *Aphanizomenon*, *Raphidiopsis*, *Dolichospermum*, *Microcystis*, *Cylindrospermopsis*, *Cuspidothrix*, *Planktothrix*, *Komvophoro*n and *Aphanizomenon-Sphaerospermopsis-Chry* complex. Non-toxic cyanobacteria included *Chroococcus*, *Planktolyngbya*, *Cyanophyta*, *Dacytlococcopsis-Raphidiopsis* complex, *Phormidium-Oscillatoria* complex, Aphanocapsa-*Aphanothece* complex, and *Merismopedia*. Taste and odor cyanobacteria were identified as *Pseudanabaena*.

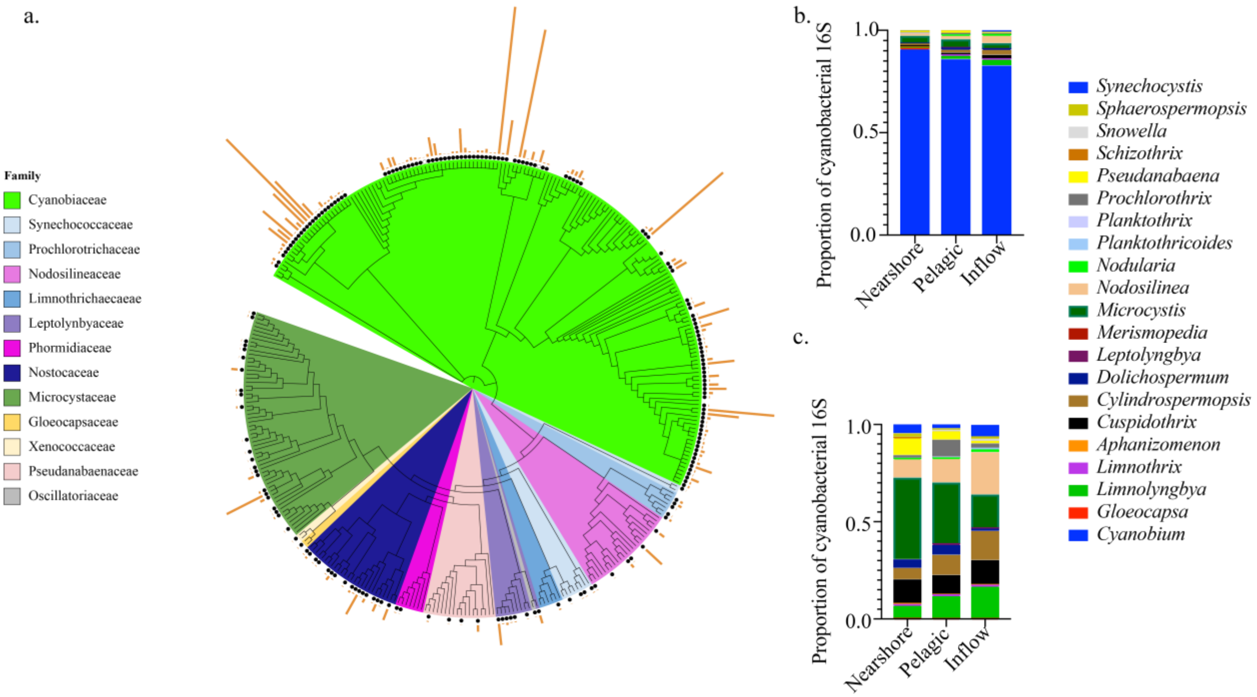

Figure S2. Analysis of cyanobacterial amplicon sequence variants (ASVs). a. Phylogenetic tree of 16S rRNA cyanobacterial ASVs from Lake Okeechobee with reference sequences from SILVA (1) database using FastTree (2) and GTR model. Black closed circles represent each ASV, and orange bars represent the average relative abundance of each ASV across all samples collected. b. The proportion of cyanobacteria genera across zones. c. The proportion of non-*Cyanobium* genera across zones.

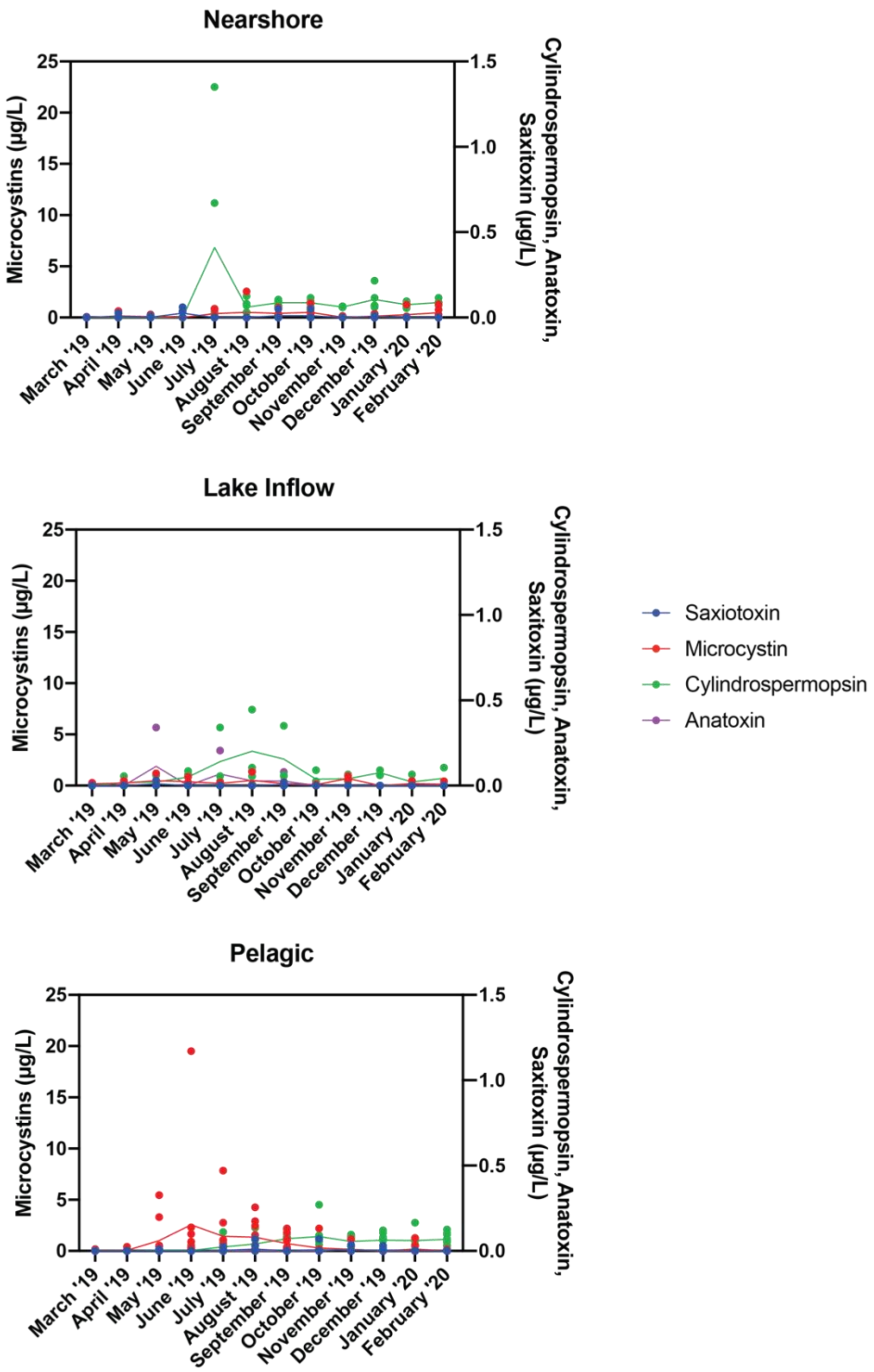

Figure S3. Cyanotoxin concentrations in Lake Okeechobee in each ecological zone. Points in graphs represent values for individual samples. The solid lines represent the average concentration of cyanotoxin for each month across all sites.

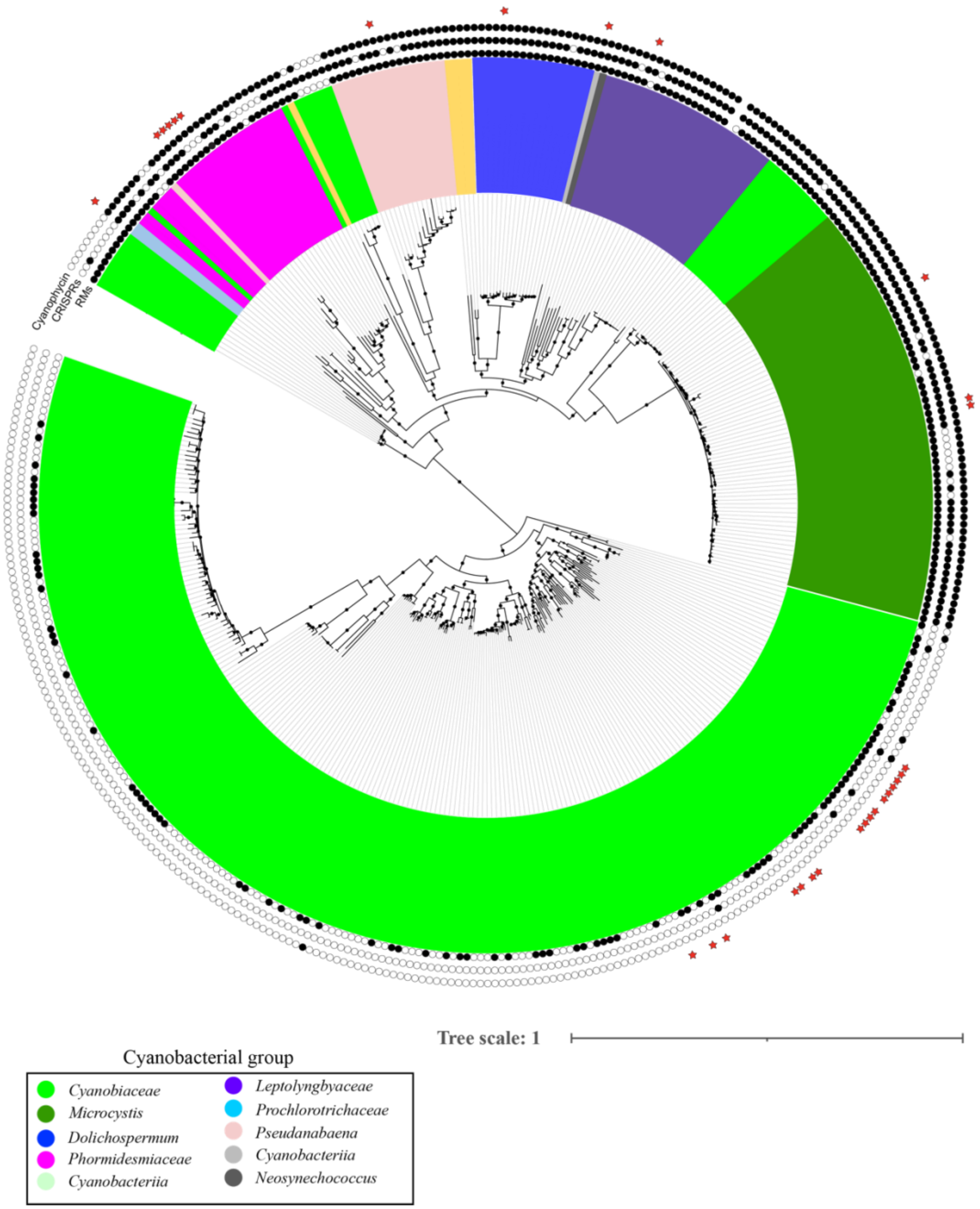

Figure S4. Phylogenetic tree generated displaying relationships between sequenced reference genomes representing the families of the cyanobacterial metagenome assembled genomes (MAGs) recovered from this study. All genomes were annotated by RAST (3) and phylogeny was determined using FastTree2 (2). The red stars indicate a cyanobacterial MAG from Lake Okeechobee. The outer rings display the presence (closed circle) or absence (open circle) of CRISPRs, restriction modification systems, and cyanophycin in each genome.

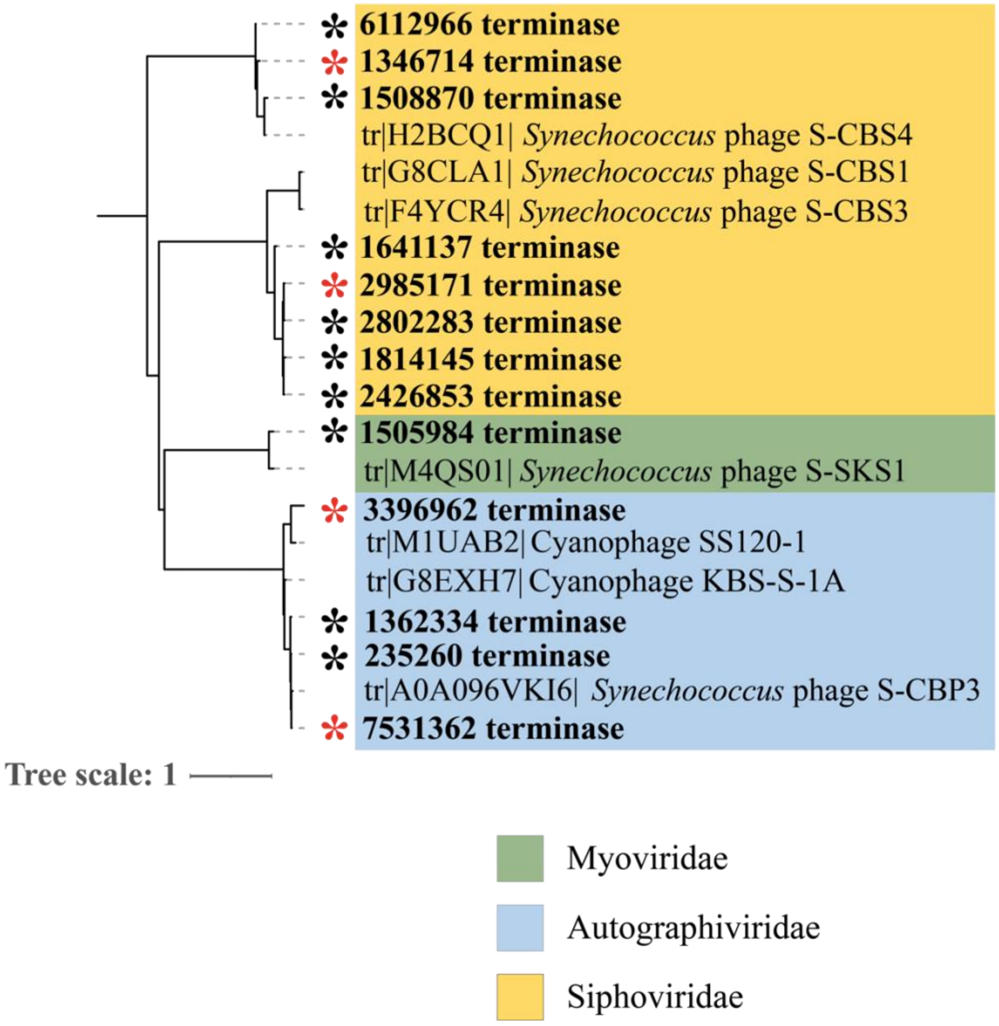

Figure S5. Maximum likelihood phylogenetic tree constructed after 1,000 bootstraps with terminase amino acid sequences from near-complete to complete phage genomes recovered from the metagenome assemblies and reference sequences. The tree was pruned so that only the closest relatives within clades are shown. Asterisks indicate which sequences represent phage found in this study and phage sequences with complete genomes recovered have asterisks in red. Taxonomic IDs for references are included in each label.

References

1. Quast C, Pruesse E, Yilmaz P, Gerken J, Schweer T, Yarza P, Peplies J, Glöckner FO. 2012. The SILVA ribosomal RNA gene database project: improved data processing and web-based tools. Nucleic acids research 41:D590-D596.
2. Price MN, Dehal PS, Arkin AP. 2009. FastTree: computing large minimum evolution trees with profiles instead of a distance matrix. Molecular biology and evolution 26:1641-1650.
3. Aziz RK, Bartels D, Best AA, DeJongh M, Disz T, Edwards RA, Formsma K, Gerdes S, Glass EM, Kubal M. 2008. The RAST Server: rapid annotations using subsystems technology. BMC genomics 9:1-15.
4. Chaumeil P-A, Mussig AJ, Hugenholtz P, Parks DH. 2020. GTDB-Tk: a toolkit to classify genomes with the Genome Taxonomy Database. Oxford University Press.
5. Parks DH, Imelfort M, Skennerton CT, Hugenholtz P, Tyson GW. 2015. CheckM: assessing the quality of microbial genomes recovered from isolates, single cells, and metagenomes. Genome research 25:1043-1055.
6. Camacho C, Coulouris G, Avagyan V, Ma N, Papadopoulos J, Bealer K, Madden TL. 2009. BLAST+: architecture and applications. BMC bioinformatics 10:1-9.
7. Sayers EW, Bolton EE, Brister JR, Canese K, Chan J, Comeau DC, Connor R, Funk K, Kelly C, Kim S, Madej T, Marchler-Bauer A, Lanczycki C, Lathrop S, Lu Z, Thibaud-Nissen F, Murphy T, Phan L, Skripchenko Y, Tse T, Wang J, Williams R, Trawick BW, Pruitt KD, Sherry ST. 2022. Database resources of the national center for biotechnology information. Nucleic Acids Res 50:D20-d26.
